# Salvage robot‐assisted radical prostatectomy with pelvic lymph node dissection for radiorecurrent prostate cancer in a patient with a previous history of rectal cancer surgery

**DOI:** 10.1002/iju5.12817

**Published:** 2024-12-10

**Authors:** Naoki Imasato, Shugo Yajima, Ryo Andy Ogasawara, Minoru Inoue, Kohei Hirose, Ken Sekiya, Madoka Kataoka, Yasukazu Nakanishi, Hitoshi Masuda

**Affiliations:** ^1^ National Cancer Center Hospital East Chiba Japan

**Keywords:** colorectal cancer, colorectal surgery, prostate cancer, prostatectomy

## Abstract

**Introduction:**

Severe adhesions render salvage robot‐assisted radical prostatectomy challenging in the treatment of patients with prostate cancer who have previously undergone colorectal cancer surgery.

**Case presentation:**

A 76‐year‐old Japanese man who had previously undergone low anterior resection for rectal cancer presented with an elevated prostate‐specific antigen level, indicating a recurrence of prostate cancer that had been treated with intensity‐modulated radiation and androgen deprivation therapies. During the salvage robot‐assisted radical prostatectomy with pelvic lymph node dissection, severe adhesions were noted between the posterior aspect of the prostate and the intestine. The adhesions were successfully dissected under digital rectal examination and transrectal ultrasound guidance.

**Conclusion:**

Salvage robot‐assisted radical prostatectomy after rectal cancer can be challenging. The use of transrectal ultrasound and digital rectal examination can facilitate the procedure. Screening for prostate cancer prior to colorectal cancer surgery could potentially allow for simultaneous resections.

**Abbreviations & Acronyms**
CRC: colorectal cancer
IMRT: intensity‐modulated radiation therapy
LAR: low anterior resection
MRI: magnetic resonance imaging
PCa: prostate cancer
PLND: pelvic lymph node dissection
PSA: prostate‐specific antigen
RARP: robot‐assisted radical prostatectomy
sRARP: salvage robot‐assisted radical prostatectomy

**Keynote message**

The concurrence of colorectal cancer and prostate cancer is increasing. Salvage robot‐assisted radical prostatectomy after colorectal cancer surgery can be challenging due to the possibility of severe adhesions. Digital rectal examination and ultrasound are useful for delineating intestinal contours during dissection between the intestines and the posterior aspect of the prostate, reducing the risk of intestinal injury.

## Introduction

The well‐documented concurrence of PCa and CRC, the most prevalent cancer types among males, necessitates the evaluation of PCa prior to CRC surgery to identify synchronous malignancies.^1^, ^2^, ^3^ With the increasing concurrence of these two types of cancer, it is possible that RARP will become more common in patients who have previously undergone CRC surgery.^4^ However, RARP in such cases can be challenging owing to the potential presence of adhesions.

Herein, we present the case of a patient with a history of previous rectal cancer surgery, who required sRARP with PLND for radiorecurrent PCa. Upon the intraoperative discovery of severe adhesions, we dissected the ones between the intestine and the posterior aspect of the prostate under the guidance of digital rectal examination and transrectal ultrasound.

## Case presentation

**Fig. 1:**
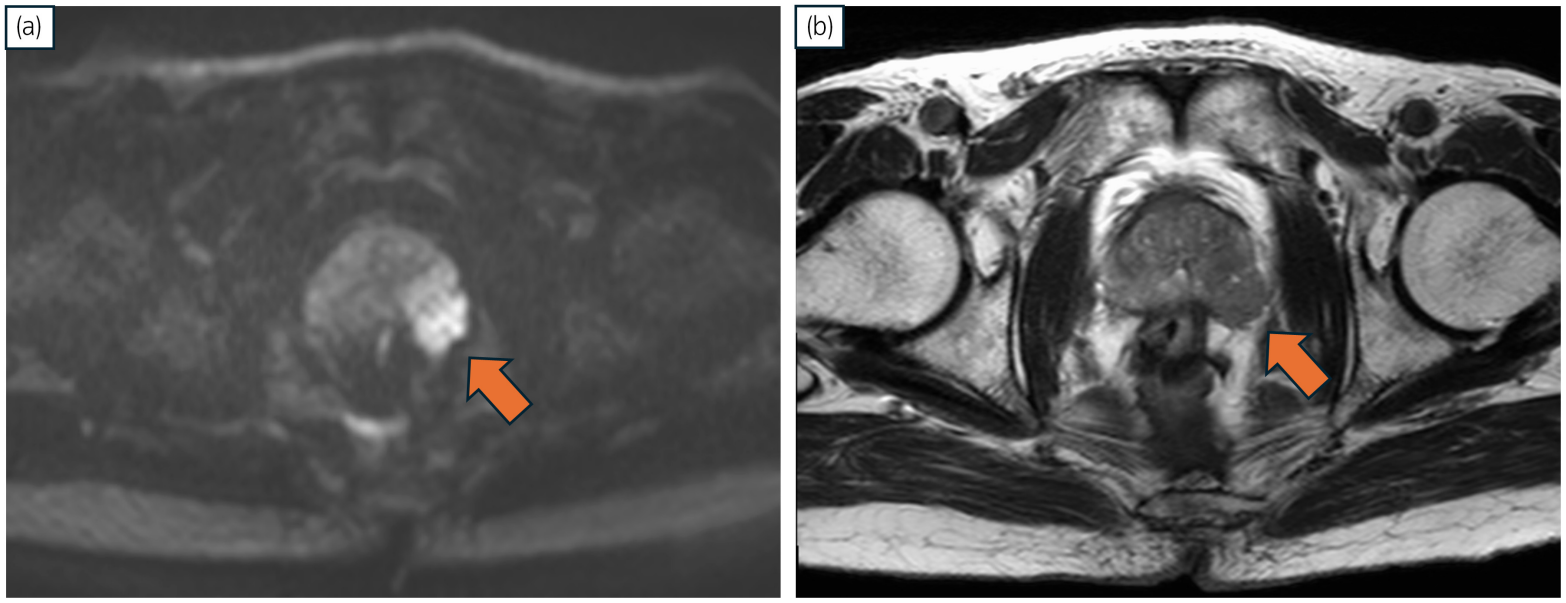
(a) Diffusion‐weighted and (b) T2‐weighted magnetic resonance images revealed extracapsular extension at the time of initial diagnosis.

**Fig. 2:**
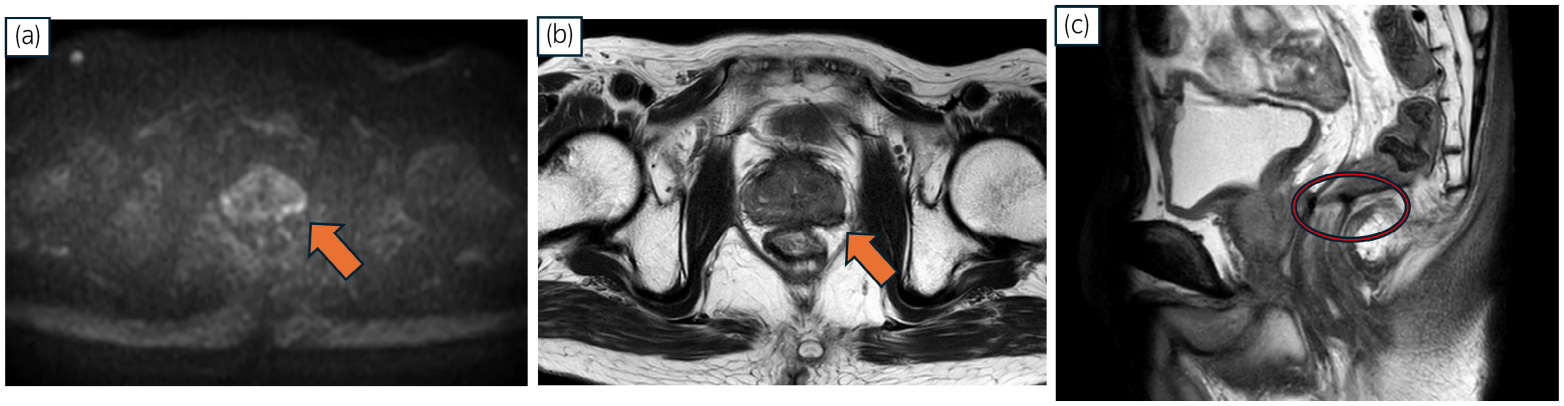
(a) Diffusion‐weighted and (b) T2‐weighted magnetic resonance images showed no malignancy at the time of recurrence. (c) Sagittal T2‐weighted image indicating the anastomosis site (red circle) behind the prostate, likely from the previous LAR.

A 76‐year‐old Japanese man with a history of open LAR for rectal cancer in 2014 was referred to our institution owing to an elevated PSA level (22.1 ng/mL) detected during a follow‐up session that same year. MRI revealed malignancy in the left peripheral zone of the prostate, indicating a diagnosis of T3a (Fig. 1). Transperineal prostate biopsy confirmed prostatic adenocarcinoma with a Gleason score of 3 + 4. Both computed tomography and bone scans showed no metastatic lesions. The patient received IMRT (76 Gy in 38 fractions) combined with androgen deprivation therapy for localized PCa in 2015. After IMRT, his serum PSA level remained stable for several years but increased again to 2.86 ng/mL in 2023. Axial MRI at the time of recurrence showed no malignant findings (Fig. 2a,b). The sagittal view revealed a region behind the prostate that appeared to correspond to the anastomosis from the LAR (Fig. 2c). Transperineal prostate biopsy revealed prostatic adenocarcinoma with a Gleason score of 4 + 3. Computed tomography and bone scans showed no metastatic lesions. On the basis of these findings, we diagnosed PCa recurrence after IMRT and decided to perform sRARP with PLND due to the patient's clinically high‐risk T3 PCa at initial diagnosis. A highly skilled surgeon performed the procedure using a Da Vinci Xi surgical system (Intuitive Surgical Inc., Sunnyvale, CA, USA) with a 6‐port intraperitoneal approach. Intraoperative findings revealed severe intraabdominal adhesions caused by the previous LAR (Fig. 2a). Owing to the difficulty and safety concerns associated with laparoscopic adhesiolysis, the surgeon made a 7 cm midline incision to divide the adhesions under direct vision. The midline rectus sheath was closed with No. 1 polydioxanone sutures, leaving sufficient space for cephalad placement of the camera port. Other trocars were placed in a standard manner after pneumoperitoneum creation. The procedure began with PLND, followed by RARP. During the latter, minimal adhesions were present at the anterior and lateral aspects of the prostate. However, severe adhesions existed between the posterior aspect of the prostate and the intestine. The surgical team carefully dissected between these structures, using transrectal ultrasound and digital rectal examination for guidance (Fig. 2b). These surgical procedures are shown in the Video S1. The operative time was 203 min, the console time was 133 min, and the estimated blood loss was 280 mL.

**Fig. 3:**
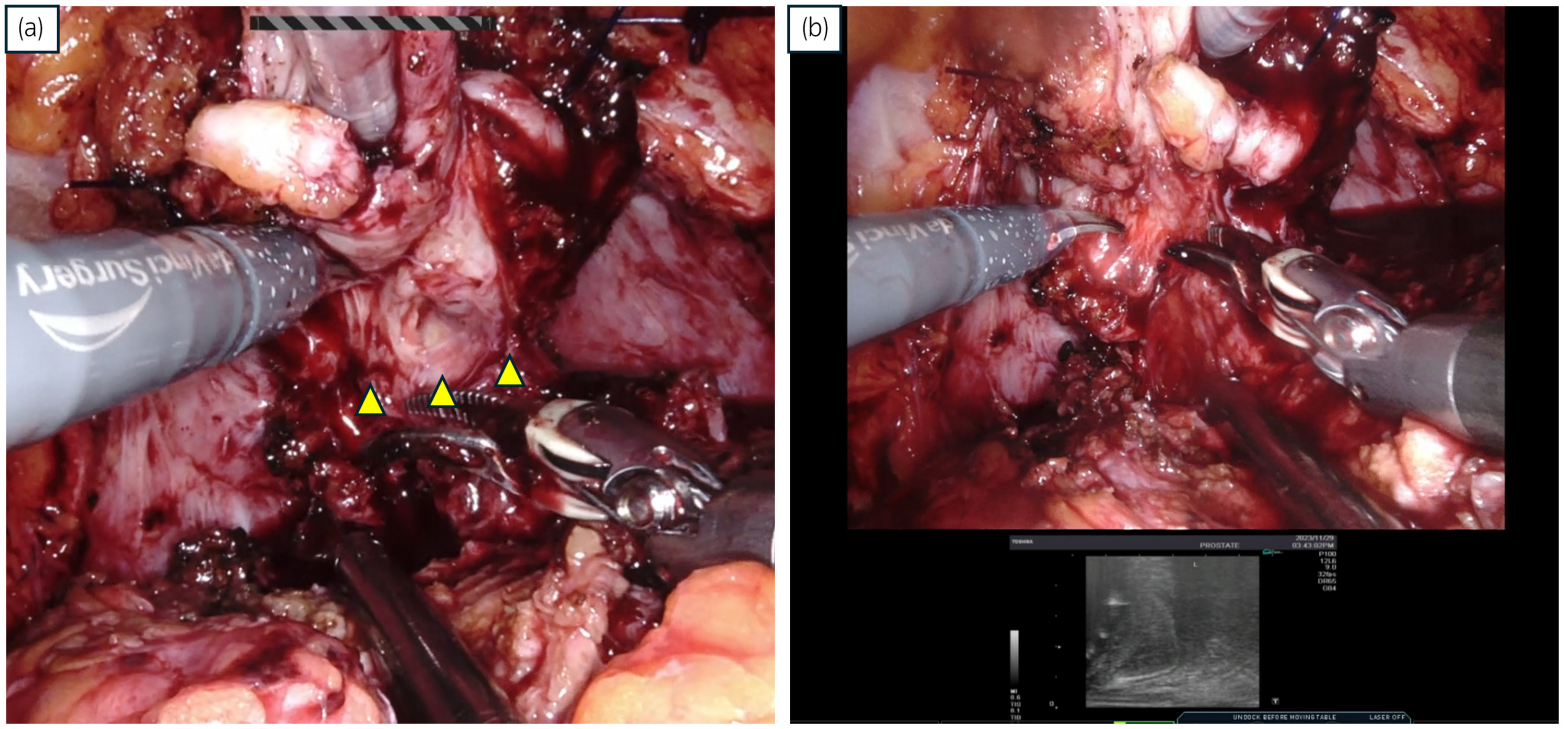
(a) Severe adhesions between the intestine and the posterior aspect of the prostate are evident. (b) Adhesions between the intestine and the posterior aspect of the prostate were dissected under the guidance of transrectal ultrasound and digital rectal examination.

**Fig. 4:**
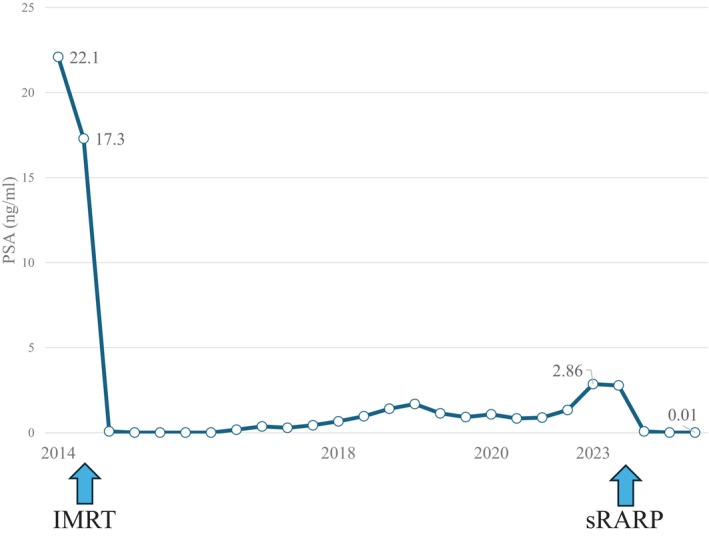
Trends in the patient's PSA levels. An initial elevation was observed in 2014. Following IMRT, the levels stabilized. A subsequent increase was detected in 2023, indicating biochemical progression. The levels remained stable after sRARP.

Urethrocystography, performed on postoperative day 6, confirmed the absence of urinary leakage at the vesicourethral anastomosis site, and the patient was discharged on postoperative day 8. No severe postoperative complications were observed. The pathology confirmed negative resection margin. At the 6‐month postoperative follow‐up, the patient had achieved pad‐free urinary continence and maintained undetectable serum PSA levels (Figs 3, 4).

## Discussion

Recurrent PCa after radiation therapy is not uncommon, with recurrence rates ranging from 20% to 60%.^2^ Salvage radical prostatectomy can improve long‐term disease‐free survival, though radiation‐induced tissue adhesions complicate dissections, especially between the rectum and prostate, increasing rectal injury risk.^5^

In this case, sRARP was performed on a patient with a previous history of rectal cancer surgery. Adhesions between the intestine and posterior prostate are a challenge in sRARP. Moreover, there are not many reports on RARP for patients diagnosed with secondary primary PCa after rectal cancer surgery. When sRARP is performed following rectal cancer surgery, the procedure can become more complex compared to sRARP after radiation therapy alone. LAR anastomoses are often directly behind the prostate, heightening the likelihood of severe adhesions. However, due to the limited number of reports, the degree of adhesions caused by the position of the anastomosis relative to the prostate remains speculative.

Men with CRC, especially those <55 years old, have a higher risk of developing secondary PCa.^4^, ^6^, ^7^ As a result, more CRC survivors require PCa treatment.^4^ Simultaneous LAR and RARP can reduce surgical time and recovery while maintaining efficacy.^8^, ^9^ Screening for PCa during CRC diagnosis could improve outcomes and reduce multiple surgeries' risks.^6^, ^8^, ^9^ In our case, screening might have enabled simultaneous resection.

With increasing CRC and PCa concurrence, more PCa diagnoses post‐CRC surgery are anticipated. For patients treated with radiation for PCa and with previous CRC surgery, complex procedures for radiorecurrent PCa are expected. The sRARP procedure is challenging owing to severe adhesions stemming from prior surgeries, necessitating effective strategies to address the issue. In our clinical setting, we used digital rectal examination and transrectal ultrasound to guide the dissection of the adhesions in the lithotomy position. The advantage of these methods is their facilitation of the ability to delineate the contour of the intestines during dissection, thereby reducing the risk of intestinal injury.

In this case, there are several limitations. First, although we performed PLND, the necessity of PLND in such cases remains debatable. As we previously reported in our case series,^10^ its necessity in the setting of PLND on salvage surgery is still under discussion. Second, the use of anti‐adhesive agents during LAR is unknown, which may have influenced the adhesions observed during RARP. More detailed patient information would be needed to determine if this had any impact on the RARP procedure. This case underscores the technical challenges associated with performing this procedure in recurrent cases. Given the intricate nature of the surgery, which demands advanced skills and precise handling, we recommend that such procedures be reserved for highly experienced surgeons. Attempting this without substantial expertise may increase the risk of complications and reduce the likelihood of successful outcomes.

## Conclusions

The incidence of sRARP after rectal cancer surgery is expected to increase, necessitating effective strategies to address this trend. Screening and early diagnosis are crucial for minimizing multiple surgeries' risks and improving outcomes.

## Author contributions

Naoki Imasato: Conceptualization; investigation; methodology; validation; software; data curation; writing – original draft; formal analysis; project administration; writing – review and editing. Shugo Yajima: Conceptualization; methodology; supervision; data curation; software; investigation; writing – review and editing; formal analysis; validation. Ryo Andy Ogasawara: Data curation. Minoru Inoue: Methodology. Kohei Hirose: Visualization; formal analysis. Ken Sekiya: Software. Madoka Kataoka: Investigation. Yasukazu Nakanishi: Writing – review and editing; supervision; methodology. Hitoshi Masuda: Writing – review and editing; supervision; conceptualization; methodology; formal analysis; validation; visualization.

## Conflict of interest

The authors declare no conflict of interest.

## Approval of the research protocol by an institutional reviewer board

Not applicable.

## Informed consent

Written informed consent was obtained from the patient.

## Registry and the registration No. of the study/trial

Not applicable.

## Supporting information


**Video S1.** Intraoperative video demonstrating key surgical techniques, including adhesion dissection and preservation of urethral length during salvage robot‐assisted radical prostatectomy after rectal cancer surgery.

## References

[1] Siegel RL , Giaquinto AN , Jemal A . Cancer statistics, 2024. CA Cancer J. Clin. 2024; 74: 12–49. 38230766 10.3322/caac.21820

[2] Kaffenberger SD , Smith JA . Salvage robotic radical prostatectomy. Indian J. Urol. 2014; 30: 429–433. 25378826 10.4103/0970-1591.142074 PMC4220384

[3] Touma NJ , Izawa JI , Chin JL . Current status of local salvage therapies following radiation failure for prostate cancer. J. Urol. 2005; 173: 373–379. 15643174 10.1097/01.ju.0000150627.68410.4d

[4] Celentano G , Creta M , Napolitano L *et al*. Prostate cancer diagnosis, treatment and outcomes in patients with previous or synchronous colorectal cancer: a systematic review of published evidence. Diagnostics 2022; 12: 1475. 35741285 10.3390/diagnostics12061475 PMC9221875

[5] Gontero P , Marra G , Alessio P *et al*. Salvage radical prostatectomy for recurrent prostate cancer: morbidity and functional outcomes from a large multicenter series of open versus robotic approaches. J. Urol. 2019; 202: 725–731. 31075058 10.1097/JU.0000000000000327

[6] Luciani LG , Mattevi D , Puglisi M *et al*. Robotic‐assisted radical prostatectomy following colo‐rectal surgery: a user's guide. J. Robot. Surg. 2022; 16: 189–192. 33743146 10.1007/s11701-021-01228-1

[7] Kim HS , Choi YJ , Shin DW *et al*. Secondary primary prostate cancer after colorectal cancer: a nationwide population‐based cohort study in Korea. J. Cancer Prev. 2017; 22: 241–247. 29302582 10.15430/JCP.2017.22.4.241 PMC5751842

[8] Maeda A , Takahashi H , Watanabe K *et al*. The clinical impact of robot‐assisted laparoscopic rectal cancer surgery associated with robot‐assisted radical prostatectomy. Asian J. Endosc. Surg. 2022; 15: 36–43. 34145964 10.1111/ases.12961

[9] Park M , Kim SC , Chung JS *et al*. Simultaneous robotic low anterior resection and prostatectomy for adenocarcinoma of rectum and prostate: initial case report. Springerplus 2016; 5: 1768. 27795910 10.1186/s40064-016-3456-y PMC5059363

[10] Yajima S , Nakanishi Y , Umino Y *et al*. Salvage robot‐assisted radical prostatectomy (sRARP) for radiation‐recurrent prostate cancer: a single‐center experience. Indian J. Surg. Oncol. 2023; 14: 361–365. 37324313 10.1007/s13193-022-01671-y PMC10267029

